# Relative importance of phenotypic trait matching and species' abundances in determining plant–avian seed dispersal interactions in a small insular community

**DOI:** 10.1093/aobpla/plv017

**Published:** 2015-03-05

**Authors:** Aarón González-Castro, Suann Yang, Manuel Nogales, Tomás A. Carlo

**Affiliations:** 1 Island Ecology and Evolution Research Group (CSIC-IPNA), C/Astrofísico Francisco Sánchez n° 3, 38206, La Laguna, Tenerife, Canary Islands, Spain; 2 Department of Biology, Pennsylvania State University, 208 Mueller Laboratory, University Park, PA 16802, USA; 3 Present address: Instituto de Ciencia Innovación Tecnología y Saberes Universidad Nacional de Chimborazo, Avenida Antonio José de Sucre, Riobamba, Ecuador; 4 Present address: Biology Department, Presbyterian College, 503 South Broad Street, Clinton, SC 29325, USA

**Keywords:** Dispersal, frugivory, mutualistic networks, oceanic islands, probability matrices

## Abstract

Network theory has provided a general way to understand mutualistic plant–animal interactions at the community level. However, the mechanisms responsible for interaction patterns remain controversial. In this study we use a combination of statistical models and probability matrices to evaluate the relative importance of species morphological and nutritional (phenotypic) traits and species abundance in determining interactions between fleshy-fruited plants and birds that disperse their seeds. The models included variables associated with species abundance, a suite of variables associated with phenotypic traits (fruit diameter, bird bill width, fruit nutrient compounds), and the species identity of the avian disperser. Results show that both phenotypic traits and species abundance are important determinants of pairwise interactions. However, when considered separately, fruit diameter and bill width were more important in determining seed dispersal interactions. The effect of fruit compounds was less substantial and only important when considered together with abundance-related variables and/or the factor ‘animal species’.

## Abstract (precis)

In this paper the authors take advantage of the simplicity of an insular community to evaluate the relative importance of species' phenotypic traits and species' abundance in determining fruit-avian disperser interactions, at both network and pairwise interaction levels. The authors innovatively include fruit nutrient compounds in fruit-avian network analyses. Although the best way to predict plant-avian interactions was based on both phenotypic traits and species abundance, the most important factor to explain these mutualistic interactions was fruit-beak size overlap, followed by species abundance and fruit nutrient compounds. This work will encourage further studies to look for similar patterns in more species-rich communities.

## Introduction

A ubiquitous mutualistic plant–animal interaction is that between fleshy-fruited plants and the fruit-eating animals that disperse their seeds (Jordano 2000). Seed dispersal interactions are complex because they involve multiple species of animals and plants, the temporal and spatial variability of such interactions (Yang *et al.* 2014) and the influence of frugivore behaviour and physiology, as well as the chemistry of fruits (Jordano 2000; Carlo and Yang 2011). Network theory has emerged as a useful tool to deal with such complexity and to search for organizational and coevolutionary patterns in community-wide plant–frugivore interactions (e.g. Bascompte *et al.* 2003, 2006; Jordano *et al.* 2003; Bascompte and Jordano 2007; Rezende *et al.* 2007; González *et al.* 2010; Mello *et al.* 2011; Aizen *et al.* 2012; Stouffer *et al.* 2012). However, the mechanisms responsible for interaction patterns in such networks (e.g. nestedness, modularity, interaction asymmetry, degree distribution) remain unclear.

Two hypotheses are available to explain how mutualistic interactions influence the structure of mutualistic networks. The first is the neutrality hypothesis (so-called abundance hypothesis), which states that observed patterns within a community are due to random species interactions. According to neutrality, probabilities of observing a plant–disperser interaction chiefly depend on the abundance of species. For example, observing both common and rare frugivores feeding on common fruiting plant species is more likely than on rare ones. This implies that abundance will be positively correlated with the level of generalization in the mutualistic interactions, i.e. highly abundant species could artificially appear as generalists that are highly connected in the mutualistic network, and rare species as more specialized (e.g. Dupont *et al.* 2003; Vázquez 2005; Vázquez *et al.* 2007; Schleuning *et al.* 2011).

On the other hand, the phenotypic traits hypothesis postulates that interaction patterns result from morphological, physiological, behavioural or evolutionary constraints that condition interaction probabilities between potential mutualistic partners (Jordano *et al.* 2003; Rezende *et al.* 2007; Santamaría and Rodríguez-Gironés 2007; Dupont and Olesen 2009; Mello *et al.* 2011; Olesen *et al.* 2011). Among phenotypic traits, the most commonly used in analyses of seed dispersal networks are the disperser bill width and fruit diameter (i.e. this will determine whether or not a seed can be swallowed and dispersed), as well as accessibility restrictions by frugivores (e.g. Rezende *et al.* 2007; Olesen *et al.* 2011; Burns 2013). However, although it has been shown that the chemical compounds of fruit can be important in determining frugivory and seed dispersal interactions (Jordano 2000 and references therein), such traits have not been used previously in network analyses. In this study we incorporate, for the first time, fruit nutritional compounds into the analysis of a frugivory network.

Some studies have demonstrated that both mechanisms (abundance and phenotypic traits) can work hand-in-hand to shape network structure (e.g. Stang *et al.* 2006; Bascompte and Jordano 2007). In this vein, Verdú and Valiente-Banuet (2011) demonstrated that facilitative interactions between plants were better explained by a combination of abundance and phylogenetic relationships than by these variables separately. Still, both hypotheses are not necessarily mutually exclusive and ecologists are beginning to examine their relative importance. Because interaction networks can be presented as adjacency matrices, Vázquez *et al*. (2009*a*) proposed using probability matrices (derived from species abundance and their spatial–temporal overlap) to assess the relative importance of abundance and phenotypic traits to determine the observed patterns of mutualistic interactions. This approach is useful to predict aggregate network parameters, but not effective in predicting pairwise interactions (Vázquez *et al.* 2009*a*).

To improve this approach, here we model pairwise interactions between fleshy-fruited plants and their avian dispersers, as a response to species’ phenotypic traits, as well as to the species’ abundance. Then we use interaction frequencies predicted by the best statistical model to build a probability matrix as proposed by Vázquez *et al*. (2009*a*) to assess the ability of that model to predict aggregate network parameters. Solving these questions will help us to understand evolutionary and ecological forces driving the assemblage of interactions in mutualistic communities (Bascompte and Jordano 2007).

## Methods

### Study area

The study was carried out in Los Adernos, a Mediterranean scrubland habitat site located in the northwest region of Tenerife (Canary Islands, UTM: 28R 317523 E/3138253 N, 220 m above sea level (a.s.l.)). The climate is Mediterranean, with mean annual rainfall ranging between 200 and 400 mm and mean temperature between 16 and 19 °C. The fleshy-fruited plant community is mainly composed of *Asparagus plocamoides* Webb *ex* Svent., *Jasminum odoratissimum* L., *Rubia fruticosa* Aiton, *Rhamnus crenulata* Aiton and *Heberdenia excelsa* (Aiton) Banks *ex* DC. There are four avian disperser species: *Sylvia atricapilla*, *S. melanocephala*, *Turdus merula* and *Erithacus rubecula*. The study was conducted in two sampling periods encompassing two whole years: from June 2008 through May 2009, and from January 2010 through December 2010.

### Seed dispersal interactions

In order to characterize the set of interactions between fleshy-fruited plants and avian dispersers, we focussed on seeds recovered from the faeces of birds captured with mist nets that were opened from dawn until dusk 2–3 days per month. We computed mist-netting effort by multiplying the mist-net length by the number of hours they were operated. Faecal samples were analysed with a dissecting scope for seeds, which were counted and identified to species level. In order to take into account interspecific differences on the number of seeds produced per fruit, we divided the number of seeds dispersed by the mean number of seeds produced per fruit. Doing so gave us a better estimation of the number of times a given disperser visit a plant species for fruits. With these data we constructed an interaction matrix based on the interaction frequency between fleshy-fruited plants and avian dispersers **[see Supporting Information]**.

Network theory has been usually applied for large and complex communities, whereas the community in this study is small (four animal and nine plant species). Small communities, however, are less prone to sample bias than large ones (Blüthgen 2010), and the reliability of studies will be greater when the more accurate is the sampling of interactions. We used an accumulation curve to prove the robustness of our sampled interactions, with a curve slope lower than 0.03 after all our 54 mist-netting sessions **[see Supporting Information]**.

### Explanatory variables

We considered eight explanatory variables associated with phenotypic traits (six variables) and abundance hypotheses (two variables) in order to explain interaction frequency between plants and animals (i.e. number of dispersed seeds). Explanatory variables for phenotypic traits hypothesis were fruit organic compounds (fibre, lipids, sugars and proteins), the identity of bird species and size overlap between fruit diameter and bill width of birds (hereafter size overlap). Although a wide variety of fruit chemical compounds may influence the choice of fruits by birds (Jordano 2000 and references therein), we selected sugars, fibre, proteins and lipids based on a study on Mediterranean avian-dispersed fruits (Herrera 1987). We decided to include the factor ‘animal species’ involved in each plant–animal interaction because species identity is important to predict animal interaction patterns (Carlo *et al.* 2003; Carnicer *et al.* 2009).

The two explanatory variables used to test for the abundance hypothesis were the product of abundance of interacting species (hereafter abundance) and temporal overlap of species phenophase length (time length which plants display fruits and bird species are present at the study site). Although phenophase length and hence temporal overlap are, to some extent, species-specific traits, they can also be considered as metrics of abundance because a species can be abundant either by producing high fruit densities and/or by being available over long time periods (Vázquez *et al.* 2009*a*). Moreover, the phenophase length of fruiting plants can also be affected by external factors to the plant such as weather conditions or the depletion of fruit crops.

#### Fruit nutrient compounds

Chemical analyses of fruits were performed by Canagrosa Laboratories (http://www.canagrosa.com/). Amount of compounds was calculated as percentage of dry mass by different methods: Kjeldahl method for proteins, gravimetric plus digestion with acid-detergent solution for fibre and Soxhlet extraction with hexane for lipids. The amount of sugars was calculated based on the remaining organic material following the equation:

(1) $$\text{sugars}=\frac{\text{NFES}\times 100}{100-\text{RH}},$$

where RH is the relative humidity of the sample and NFES (nitrogen-free extractive substances) is calculated as follows:

(2) NFES=100−RH−proteins−lipids−fibre.

#### Animal species

We accounted for the animal species identity as a factor to explain the interaction frequency. Although quantification of animal traits exists for the species studied here (Herrera 1984; Jordano 1987), our system is unfortunately too small (36 interactions) to support models with these additional explanatory variables. We decided to give priority to use fruit nutrient compounds because animal traits have been previously used in some extent on network analyses, whereas fruit nutrients have not yet.

#### Size overlap

To account for individual variability of fruit and bill size, we decided to use the range (mean ± SD) in both fruit diameter and bird gape width instead of just comparing their mean values. For each pair of species, we calculated the percentage of range of fruit diameter that was equal or smaller than the maximum value of the bird gape width. For example, if the diameter of a fruit species ranges from 7.0 to 8.0 mm (variation range = 1.0 mm), then the resulting overlap with the gape of a bird that ranges between 9.0 and 10.0 mm would be 100 %. However, for a bird with a gape width of 6.0–7.5 mm, the size overlap is 50 %, because only half of the fruit variation range (0.5 out of 1 mm) could be ‘swallowed’ by this second bird. For those interactions with pairs of species which size overlap was 0 % we arbitrarily establish a size overlap of 1 × 10^−5^.

#### Abundance and temporal overlap

The variable abundance is the product of abundance of the interacting species. To assess fruit abundance we used 20 plots of 5 m^2^ randomly placed. We visited every plot monthly and estimated the number of fruits per metre square for every plant species (visual counting method, Chapman *et al*. 1992). We estimated the cumulative abundance after the two study years and then calculated the relative fruit abundance for every plant species as the percentage of fruits of each species from the total community-wide fruit crop. Bird abundance was estimated by using a simple mixed effect regression analysis for every 100 h of sampling: [Individuals × m^−2^ = 2.15 + 4.117 × (100 × C); *P* = 0.001; *N* = 152], where *C* is the number of captured birds per unit of effort. To build this regression we used unpublished data (A.G.-C.) from the same study area. Censuses were performed twice per month. Therefore, we considered the date as a random effect factor to avoid an effect of temporal pseudo-replication. To ensure bird detectability in censuses, a band 25 m wide was surveyed, where all individuals (seen or heard) were counted. We consider that all disperser birds had equivalent capture probability in mist nets because shrubs mostly dominate the study site, and frugivorous birds have same movement patterns, between shrubs, where mist nets where placed.

Every 15 days we recorded the presence of species of fruits and birds to obtain the length of species phenophase. The temporal overlap is defined as the percentage of days with respect to the whole study period (i.e. 730 days) that pairs of species coincided in the study area. For example, if a plant species fruited for 60 days, and a bird species was present in the study area for 60 days, but fruit and bird species coincided for only 30 days, then they had 4.11 % of temporal overlap (30 out of 370 days).

### Modelling interaction frequency between pairs of species

We modelled the log-transformation of interaction frequency (estimated as explained above) as a response to the explanatory variables by using a generalized least squares (GLS) model in the *nlme* package (Pinheiro *et al.* 2009) implemented in R 2.11 (R Development Core Team 2015). The variance was not homogeneous and changed with the predictor ‘size overlap’, thus the GLS model allowed us to work with a normal error distribution and taking into account the variance structure by using the function ‘varFixed’, implemented in the *nlme* package (Zuur *et al.* 2009).

As our study system was small, with only 36 plant–frugivore interactions, we had to build different models with different subsets of explanatory variables to avoid model over fitting (*sensu* Burnham and Anderson 2002). Therefore, we used different combinations of phenotypic traits and abundance variables in different models **[see Supporting Information]**. By doing so, each statistical model included only a maximum of five explanatory variables (including main effects of variables and/or their statistical). In models that we could not include all fruit compounds together, we separated them into two different sets: one included ‘non-energetic’ compounds (fibre and proteins) and the other included ‘highly energetic’ compounds (sugars and lipids).

We ranked models according to the AIC value and computed the Akaike's weight as an estimation of the probability of a given model to be the best candidate model explaining the observed interactions (Burnham and Anderson 2002). To evaluate the importance of different explanatory variables we used a multi-model inference, based on the sum of Akaike's weight of each model where each explanatory variable appeared (Burnham and Anderson 2002). Different variables appeared in a very different number of models (e.g. size overlap appeared in three models, whereas ‘animal species’ appeared in 14 models), based on the natural history of the fruiting plants and animals rather than their statistical importance. For such a reason, we averaged the sum of Akaike's weight by dividing it by the total number of models where each variable appeared.

### Prediction of aggregate network parameters

With the interaction frequency predicted by the best statistical model (that showed the lowest AIC value) we created an expected interaction matrix. Subsequently, we normalized this matrix by dividing their elements by their total number of predicted interactions to obtain a probability matrix. With this probability matrix, we used simulations based on the approach of Vázquez *et al*. (2009*a*) to assess the capacity of our best model to predict different aggregate network parameters: connectance (proportion of realized interactions respect to total cells in the interaction matrix), interaction evenness (which is a Shannon index proposed by Tylianakis *et al.* 2007; the higher the index, the more evenly distributed are interactions in the matrix), nestedness (the degree to which specialists interact with proper subsets of the species that generalists interact with) and interaction asymmetry for fruits and for dispersers (Vázquez *et al.* 2007, 2009*a*). We performed 1000 randomizations for the model (i.e. the probability matrix), calculated the mean and 95 % confidence interval of each parameter and assessed if the observed value for each parameter in the interaction matrix recorded at Los Adernos fell within such confident interval.

We had problems in simulating nestedness values with the temperature algorithm proposed by Rodríguez-Gironés and Santamaría (2006), perhaps due to small size of our interaction matrix. Therefore, we used the Nestedness metric based on Overlap and Decreasing Fill (NODF; Almeida-Neto *et al.* 2008) and ‘weighted nestedness’ (Galeano *et al.* 2009) algorithms. All the analyses were run with the R-code provided by Vázquez *et al*. (2009*a*) but with modifications to include NODF and weighted nestedness measures as implemented in the *bipartite* (Dormann *et al.* 2009) package of R statistical software (R Development Core Team 2015).

## Results

### Statistical models

We created 24 statistical models, containing each explanatory variable separately, as well as different combinations of them **[see Supporting Information]**. The best model to explain plant–bird interactions combined both species phenotypic traits and species abundance. This model (AIC = −15.00) included the factor ‘animal species’ and variables related with species matching (size overlap between fruit diameter and bird bill width, temporal overlap of species phenophase length and the product of species abundance). The second best model (AIC = −4.57) included some fruit compounds (fibre and proteins), the factor ‘animal species’ and both abundance-related variables (temporal overlap and species abundance) **[see Supporting Information]**. The null model (that with only the intercept) was better than three models based only on species abundance, size overlap and ‘size overlap × animal species’, respectively **[see Supporting Information]**.

Considering the averaged sum of Akaike's weight, the most important variable was size overlap between fruit diameter and bird bill width, followed by species temporal overlap, species abundance and the factor ‘animal species’ (Table 1). However, the difference between temporal overlap and species abundance was not significant (the order of magnitude of that difference was 1 × 10^−15^). According to the Akaike's weight, fruit nutrient compounds were less important variables (Table 1). However, models that combined both fruit compounds with the identity of animal species and/or variables related with abundance were among the best fitted **[see Supporting Information]**.

**Table 1.** Relative importance of each explanatory variable to determine fruit–avian disperser interactions in Los Adernos (Northwest of Tenerife Island). Variables are ranked from most important to less important according to the averaged sum of Akaike's weight, *w*_i_ (Burnham and Anderson 2002). The higher the value, the more important the explanatory variable is. As the number of models in which each variable appears is established by our knowledge of fruit and bird natural history, and not because of statistical reasons, we decided to use the averaged sum of Akaike's weight.

Explanatory variable	Number of models in which variable appears	Averaged sum of *w_i_*
Size overlap	3	0.333333333
Temporal overlap	7	0.143707147
Species abundance	7	0.143707147
Animal species	14	0.072078903
Fibre	5	0.001181644
Proteins	5	0.001181644
Sugars	5	0.000568973
Lipids	5	0.000568973

**Figure 1.**
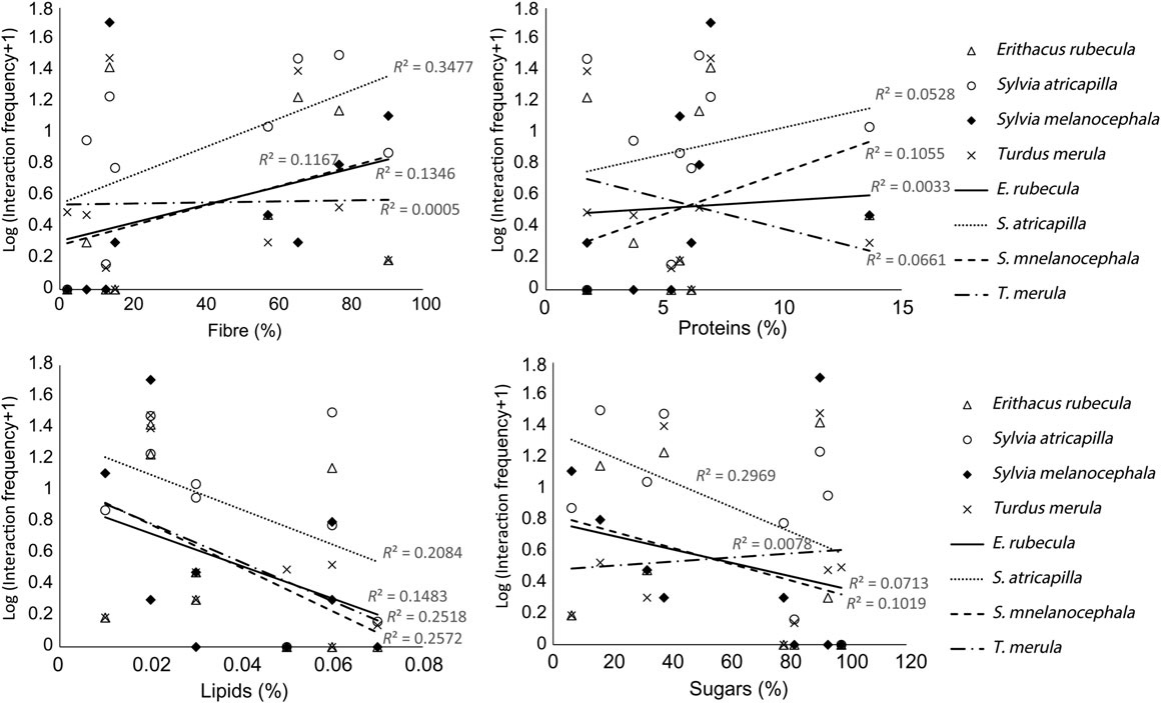
Relationship between the content of different fruit nutrient compounds and interaction frequency in Los Adernos. The figure shows the relationship with different avian disperser species. For each species (different lines) the fit (*R*^2^) is shown.

**Figure 2.**
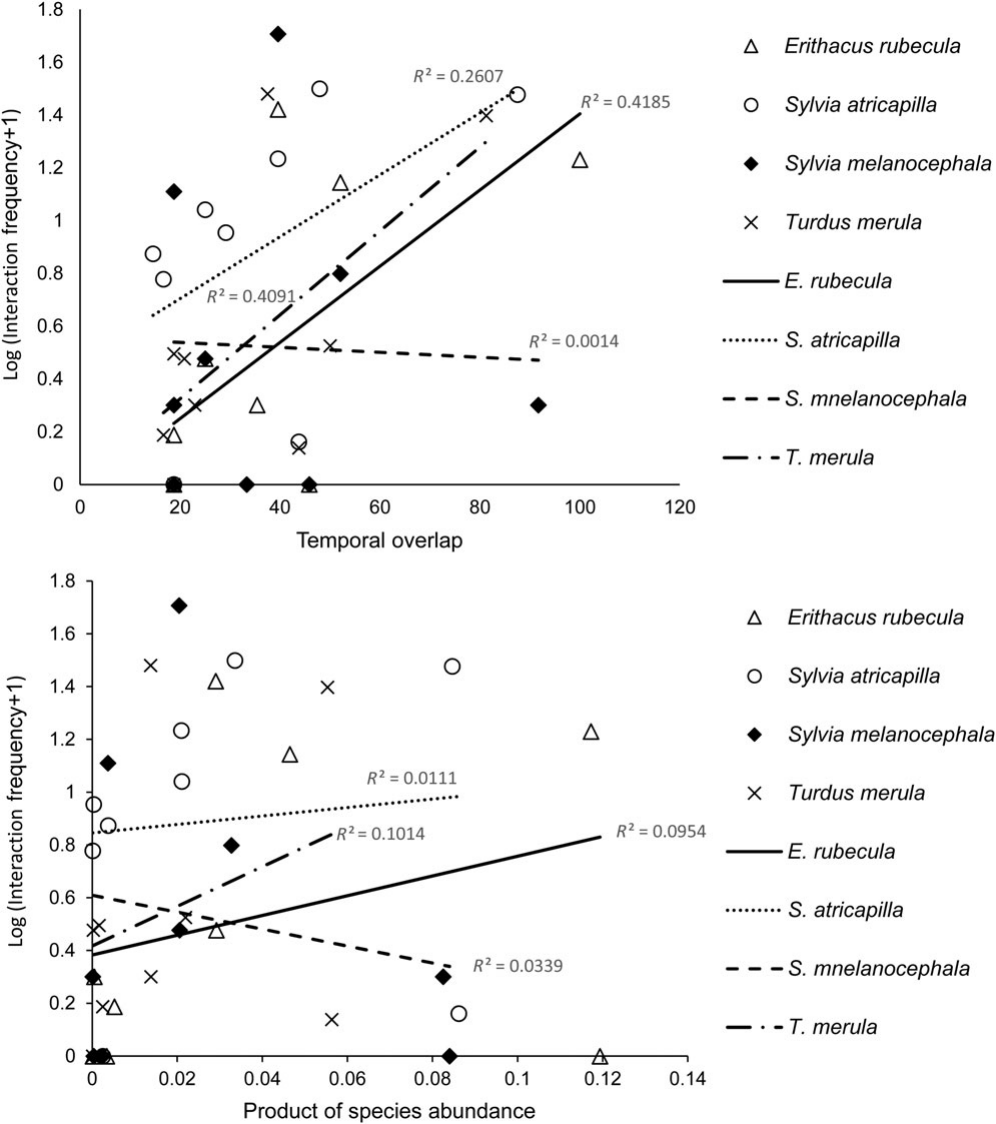
Relationship of species phenophase temporal overlap, and product of species abundance with interaction frequency in Los Adernos. The figure shows the relationship with different avian disperser species. For each species (different lines), the fit (*R*^2^) is shown.

In general, birds dispersed more frequently plant species with a lower amount of sugars and lipids in their fruits, with the exception of *T. merula*, which tend to select those fruits with higher sugar content (Fig. 1). Birds, with the exception again of *T. merula*, more often dispersed fruits with higher protein content. Curiously, fibre-rich fruits, which are of low digestibility, also had, in general, a high dispersal frequency (Fig. 1). Interaction frequency was also higher for pairs of species with a higher product of their abundance and higher temporal overlap, with the exception of *S. melanocephala* (Fig. 2).

With respect to aggregate network parameters, the probability matrix based on the best statistical model was able to predict only nestedness, based on both ‘NODF’ and ‘weighted nestedness’ algorithms (Table 2).

**Table 2.** Observed and simulated (mean and 95 % confident interval) values of six network parameters. Network parameters with observed value that coincide with confidence interval of simulation are in bold.

Network parameter	Observed value	Mean value	Lower limit	Upper limit
Connectance	0.7777778	0.9170556	0.8611111	0.9444444
**Nestedness (NODF)**	**41**.**6666667**	**30**.**0674603**	**16**.**6666667**	**48**.**8095238**
**Weighted nestedness**	**0**.**3467869**	**0**.**1300832**	**−0**.**1311452**	**0**.**4306871**
Interaction evenness	0.8341048	0.8738392	0.8471696	0.899341
Interaction asymmetry for birds	−0.2723253	−0.1723619	−0.2142811	−0.1448604
Interaction asymmetry for plants	0.1602762	0.148106	0.1422437	0.15625

## Discussion

Our study shows that the best way to understand pairwise interactions of the plant–frugivore network in the scrublands of Tenerife is using both the phenotypic traits and the abundance of species. Previous studies in other sites and mutualistic interactions (e.g. pollination) have reached similar conclusions (Stang *et al.* 2006; Bascompte and Jordano 2007; Verdú and Valiente-Banuet 2011), but our study stands out in showing that matching of two phenotypic traits (fruit diameter and bird bill width) is a stronger determinant of mutualistic interactions than species’ abundances (Table 1). Our best model was able to predict only one network parameter, nestedness (NODF and ‘weighted nestedness’), which is in contrast to previous studies able to predict more parameters (Vázquez *et al*. 2009*a*; Verdú and Valiente-Banuet 2011). However, it is important because nestedness has been proposed to be an important structural feature determining species coexistence and diffuse coevolution (Bascompte *et al*. 2003). Therefore, our results support that nestedness may be strongly influenced by both fruit-bill matching, as well as by species abundance as previously proposed (e.g. Vázquez 2005; Rezende *et al*. 2007).

Previous studies have demonstrated that an important trait related to seed dispersal frequency is the overlap between fruit diameter and bird gape width (e.g. Rezende *et al.* 2007; Olesen *et al.* 2011), which is in accord with our best model. Increasing the size overlap between the bill widths and fruit diameters increases the probability of a successful seed dispersal interaction between a bird–plant species pair (e.g. Pratt and Stiles 1985; Wheelwright 1985; Jordano 1987; Levey 1987). Size restriction could explain why in this community, some small bird species depended heavily on smaller fruits of low digestibility (e.g. *S. melanocephala*—*R. crenulata*) than on more profitable but larger fruits (e.g. *Tamus edulis*, with a 97.87 % of sugars). This size restriction could also explain why the smallest passerine (*S. melanocephala*) has very few interactions with the large-fruited *H. excelsa* (Fig. 2), despite these two species having a high temporal overlap and high abundances.

Although the importance of fruit chemistry in mediating plant–frugivore interactions has been amply demonstrated (Jordano 2000), this study is the first to include them as part of structural analyses of a mutualistic network. We found some relationship between fruit nutrient amount and interaction frequency (Fig. 1). However, fruit compounds were weak predictors of fruit–bird interaction frequency when considered independently (Table 1; and see low values of *R*^2^ in Fig. 1). On the other hand, six out of the seven best models included combinations of fruit compounds with the identity of animal species and/or species abundance **[see Supporting Information]**. This suggests that importance of fruit compounds in determining fruit–bird interactions should be considered in a global context of additional ecological factors.

In general, small birds dispersed plant species with fruits of low digestibility and profitability (i.e. low content of sugars and lipids and high content of fibre) more frequently, whereas *T. merula* tended to show the opposite pattern (Fig. 1). This result makes sense if we consider that small disperser birds of the thermophilous scrublands (*E. rubecula*, *S. atricapilla* and *S. melanocephala*) are characterized by having fruit-dominated diets and short gut-passage times (see Herrera 1984 for same bird species). Thus, when birds with short gut-passage times consume fruits of low digestibility, they would need to increase the rates of fruit intake to maintain their energy and nutrient assimilation balance (Barboza *et al.* 2009), whereas birds with long gut-passage times (*T. merula* in our case) would not need to increase the intake rate because fruit pulp remains longer in the gut (Barboza *et al.* 2009).

Our results also confirm the abundance hypothesis because of the positive relationship between the frequency of seed dispersal and the product of species abundances and their temporal overlap, with the above-mentioned exception of the *S. melanocephala–H. excelsa* interaction (Fig. 2). According to the averaged sum of Akaike's weights, temporal overlap and species abundance were more important than fruit chemical compounds (Table 1). Thus, as the product of species abundances and/or their temporal overlap increases, the more likely it is that species will interact with each other. According to previous studies (e.g. Dupont *et al.* 2003; Vázquez 2005; Vázquez *et al.* 2007; Schleuning *et al.* 2011), species abundance (as number of individuals) should be sufficient by itself to explain fruit–bird interactions. However, like for phenotypic traits, a greater importance of species abundance (and temporal overlap) emerged from four models where abundance-related variables appear jointly with other phenotypic traits **[see Supporting Information]**.

Although the importance of temporal overlap and species abundance was not significantly different (Table 1), the model solely based on temporal overlap of species’ phenophase fit better than the model only based on the product of species’ abundance **[see Supporting Information]** (Fig. 2). This finding is in accordance with previous studies on mutualistic networks (Olesen *et al*. 2008; González-Castro *et al*. 2012*b*). We have to note that although we consider temporal overlap as an abundance-related variable, species’ phenophase length is, in some extent, a species-specific trait (Olesen *et al*. 2008; González-Castro *et al*. 2012*b*). Therefore, the importance of species’ temporal overlap for plant–disperser interactions might be considered as an influence of phylogeny of species.

We can think of at least two explanations for the relatively weak effect of species abundance on plant–disperser interactions in our community, when compared with the effect of temporal overlap. One could be due to the small size of this community (four animal and nine plant species). Larger communities invariably have a higher potential number of interactions, which makes them more difficult to sample appropriately (Blüthgen 2010). In contrast, in small communities sampling community-wide interactions is more precise. For example, comparing two closely related Mediterranean habitats, González-Castro *et al*. (2012*a*) found that the effect of abundance on interaction asymmetry was lower in the small-sized community than in the large one. Another possibility is that the method used to estimate species abundances affects the outcome of the models (Vázquez *et al*. 2009*b*). Previous studies have used interaction frequency as a measure of abundance (e.g. Vázquez *et al.* 2007; Schleuning *et al.* 2011). Thus, there is an obvious lack of independence between the response (interactions) and the predictor variable (abundance). But, in this study, we measured species abundance independently of the animal–plant interaction using captures and censuses. In this sense, our abundance estimates are uncorrelated to our interaction data, and thus more appropriate than those used by previous studies. We suggest that future studies should use independent estimators of species’ abundances when trying to assess the effects of abundance on interaction patterns.

The first six models in our ranking included the factor ‘animal species’ **[see Supporting Information]**. The inclusion of this factor, and/or its interaction term with fruit compounds or abundance-related variables, generally improved the equivalent models in which ‘animal species’ was excluded **[see Supporting Information]**. Importance of interaction terms between fruit compounds and ‘animal species’ reveals the relevance of some animal phenotypic traits in determining fruit-disperser interactions, and suggests that different animal species might respond to fruit nutrients in different ways (Jordano 2000). Therefore, our results support the assertion of Jordano (2000) that: ‘the profitability of a given fruit should be examined in the context of an interaction with a particular frugivore species’.

The model including the statistical interaction ‘animal species × species abundance’ slightly improves the fit of the model with respect to the model only based on species abundance (AIC = 375.124 and 376.489, respectively) **[see Supporting Information]** (see also different bird responses in Fig. 2). This result is consistent with interspecific differences in the capacity of birds to respond to changing fruit abundances (Carnicer *et al.* 2009). This species-specific response of birds to fruit abundance makes it more difficult for abundance to determine community-wide interactions by itself.

## Conclusions

Although fruit-bill size overlap seems to be the most important variable when considered independently, both species abundance and phenotypic traits were important in determining fruit–bird interactions. The small community size (36 interactions) constrained us to use different subsets of explanatory variables in different models. However, species abundance and phenotypic traits are inseparable in a community. Therefore, the approach we used will be useful to examine more diverse communities, but using more realistic models (i.e. including all explanatory variables together). Although obtaining detailed phenotypic data in larger communities is challenging, it will allow a better understanding of ecological organization and coevolutionary processes shaping mutualistic plant–animal communities.

## Sources of Funding

A.G.-C. benefited from a JAE-PRE fellowship from the Consejo Superior de Investigaciones Científicas (Spain). The National Science Foundation under a grant awarded in 2008 funded S.Y. This work was financed by the Spanish Ministry of Science and Education project (CGL2007-61165/BOS) and is also framed within project GGL2010-18759/BOS, supported by FEDER funds from the European Union.

## Contributions by the Authors

T.A.C. and A.G.-C. conceived the idea. M.N. and A.G.-C. carried out the fieldwork, S.Y. and A.G.-C. analysed the data. S.Y., M.N., T.A.C. and A.G.-C. wrote the paper.

## Conflict of Interest Statement

None declared.

## Supporting Information

The following additional information is available in the online version of this article –

**Table S1.** Interaction frequency between plants (columns) and animals (rows) recorded at the study site.

**Figure S1.** Accumulative curve of interactions recorded at the study site against the sampling effort (mist-netting sessions).

**Table S2.** Candidate statistical models, ranked according to their AIC value.
